# Effects of urban green space habitats and tree species on ectomycorrhizal fungal diversity

**DOI:** 10.1038/s41598-024-74448-8

**Published:** 2024-10-25

**Authors:** Qian-Cai Lin, Ying-Qing Cen, Ming Xu, Dan-Dan Jiang, Jian Zhang

**Affiliations:** 1 Key Laboratory of Plant Resource Conservation and Germplasm Innovation in Mountainous Region (Ministry of Education), Collaborative Innovation Center for Mountain Ecology and Agro-Bioengineering (CICMEAB), College of Life Sciences, Guizhou University, Huaxi District, Guiyang, 550025 Guizhou China; 2 Institute of Fungal Resources, College of Life Sciences, Guizhou University, Institute of Agricultural Bioengineering, Guizhou University, Guiyang, 550025 Guizhou China

**Keywords:** Diversity, Ectomycorrhizal fungi, Greening trees, Urban greenspace habitats, Urbanization, Ecology, Ecology, Environmental sciences

## Abstract

Ectomycorrhizal fungi (EMF) are key symbiotic microbial components for the growth and health of trees in urban greenspace habitats (UGSHs). However, the current understanding of EMF diversity in UGSHs remains poor. Therefore, in this study, using morphological classification and molecular identification, we aimed to investigate EMF diversity in three EMF host plants: *Cedrus deodara* in the roadside green belt, and *C. deodara*, *Pinus massoniana*, and *Salix babylonica* in the park roadside green belt, in Guiyang, China. A total of 62 EMF Operational Taxonomic Units (OTUs) were identified, including 13 EMF OTUs in the *C. deodara* roadside green belt, and 23, 31, and 9 EMF OTUs in the park green belts. *C. deodara*, *P. massoniana*, and *S. babylonica* were respectively identified in park green belts. Ascomycota and Basidiomycota were the dominant phylum in the EMF communities in roadside and park green habitat, respectively. The Shannon and Simpson indexes of the *C. deodara* EMF community in the park green belt were higher than those in the roadside green belt. EMF diversity of the tree species in the park green belt was *P. massoniana* > *C. deodara* > *S. babylonica*. Differences in EMF community diversity was observed among the different greening tree species in the UGSHs. UGSHs with different disturbance gradients had a significant impact on the EMF diversity of the same greening tree species. These results can be used as a scientific reference for optimizing the design and scientific management of UGSHs.

**Supplementary Information:**

The online version contains supplementary material available at 10.1038/s41598-024-74448-8.

## Introduction

Urbanization has changed the human living environment and played a “filter” role to microbial diversity^1,2^. Often, alien plant species are introduced in urban areas to increase the beauty of urban landscapes, leading to ecosystem convergence. This convergence phenomenon leads to biodiversity decline, and homogenization of habitats lead to homogenization of urban soil microorganisms^3^. A reduction in contact with natural biodiversity weakens the microbiome and immune system of the human body, thereby affecting human health. Thus, rapid urbanization negatively impacts microbial biodiversity and consequently, human health^4–7^. Exposure to diverse urban greenspace habitats (UGSHs) can reduce blood pressure, relieve pain, and reduce mortality^8–10^. Although the mechanisms associated with these positive effects remain unclear, the effects may be due to the interaction of plants and soil microbial communities with humans and the soil microbiome can transfer to the residents, altering human microbiome composition, influencing immune function and health outcomes^11–13^. Therefore, it is necessary to improve our understanding of soil microbial diversity among different UGSHs.

UGSHs allow pollution degradation and remediation for human survival^14,15^, water resource management, carbon maintenance, nutrient cycling, and a series of basic ecosystem services^16–18^, such as promoting biochemical cycles and soil processes^19,20^; therefore, they are key in modern urban ecosystems^21^. “The microbiome rewilding hypothesis” proposes that microbial diversity in urban green spaces can effectively improve urban population health by providing a “natural” microbiome to urban residents^22,23^. However, owing to environmental characteristics, artificial management, and maintenance types, different UGSHs differ considerably in structure and function. Urban roadside and park green belts are common UGSHs types. Roadside green belts promote isolation, safety, and ecological protection; these soils become relatively isolated habitats surrounded by concrete fences and are subject to multiple disturbances (for example, automobile exhaust emissions, dust fall, road management, and maintenance), which can influence soil enzyme activity and organic carbon content, affecting soil microbial diversity^24,25^. Urban parks provide important ecological services such as air purification, climate regulation, environment beautification, and physical and mental health promotion of residents^26,27^. Parks are often affected by human activities, and the soil surface is trampled and compacted^28,29^, which affects soil pore connectivity, permeability, air permeability, temperature, rooting space, nutrient flow, and biological activity^30–32^. Human disturbance is the main factor affecting soil microbial diversity^33,34^. Soil microbiomes in UGSHs play an important role in the sustainable and stable development of urban ecological environments by alleviating psychological pressure and physical health^27^. Therefore, strengthening the monitoring, evaluation, and research of soil microorganisms in UGSHs is of great scientific and practical significance.

Ectomycorrhizal fungi (EMF) play an important role in maintaining biodiversity and plant community succession^35^. Pinaceae, Fagaceae, Salicaceae, and other major tree species are EMF host plants that are widely distributed in most forest ecosystems^36^. EMF help host plants absorb nutrients (nitrogen, phosphorus, potassium, and other elements) and alleviate heavy metal pollution stress and antagonize diseases^37,38^. Some EMF can form fruiting bodies during the completion of their life cycle and are often used as indicators of changes in UGSHs^39^. EMF allows for rapid establishment or promotion of early tree species colonization in disturbed areas^40^. Considering that many ectomycorrhizal host trees such as *Pinus*, *Populus*, and *Salix* are distributed in cities, UGSHs are characteristic of habitat isolation and long-term artificial perturbations (for example, exposed pollutants, artificial disturbance, and management activities) and obstruct the interspecies interactions of fungi, resulting in lower EMF diversity in UGSHs^41,42^. Therefore, a deeper understanding of EMF diversity in UGSHs will allow for improved scientific management.

A large majority of studies on EMF diversity has focused on natural ecosystem habitats such as different forest types and grasslands^43,44^, reporting changes in soil microbial communities^45^, whereas few studies have focused on EMF diversity, in UGSHs. Therefore, our research focused on the EMF diversity in UGSHs. In this study, we selected *C. deodara*, *P. massoniana*, and *S. babylonica* in the urban park roadside green belt and *C. deodara* in the urban roadside green belt as the research objects. We compared the effects of different habitats (park and roadside green belt) on the same EMF host plant and the same habitat (park roadside green belt) on the EMF diversity of different host plants. We hypothesized that, under different UGSHs: (1) EMF diversity would differ among different tree species within the same environment; and (2) EMF diversity would vary for the same tree species under different environments. The present study aimed to provide a scientific reference for optimizing the design and scientific management of UGSHs.

## Results

### EMF infestation rate

**Fig. 1:**
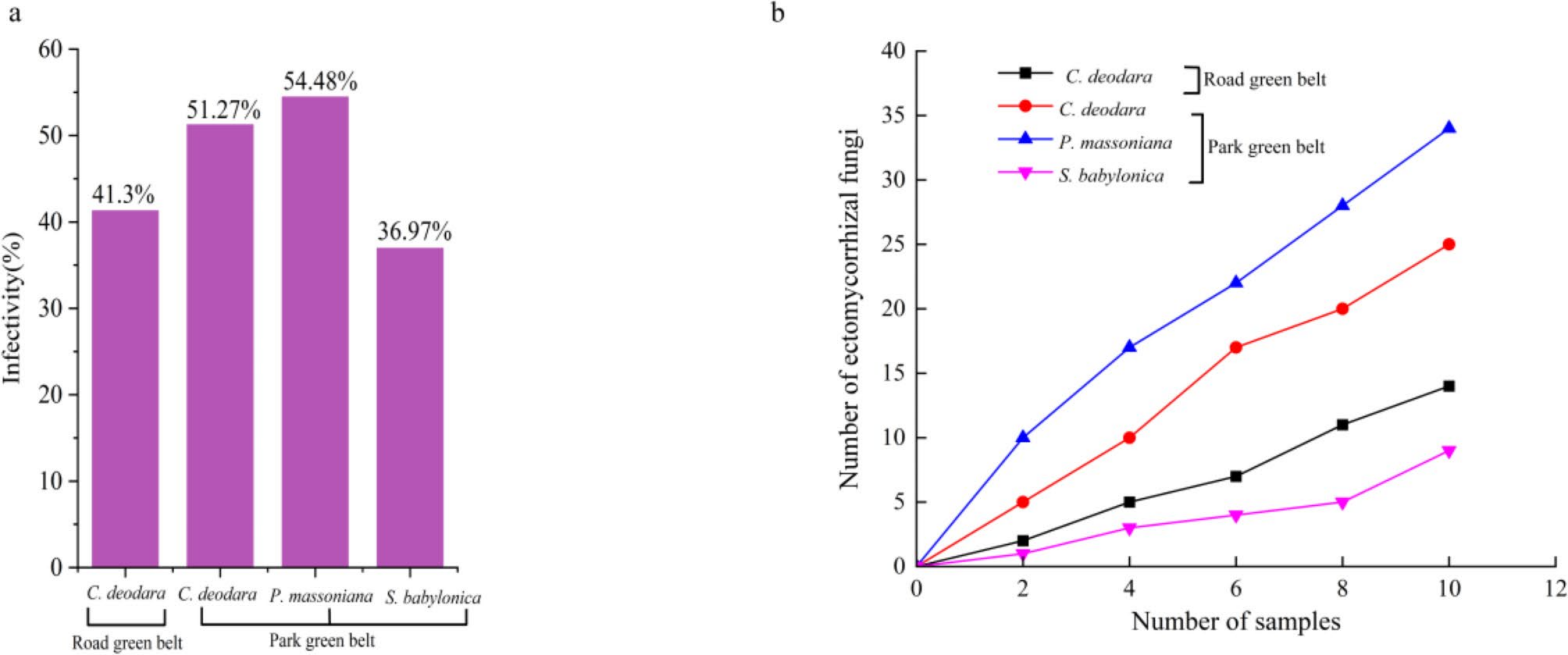
Ectomycorrhizal fungi (EMF) infection rate (**a**) and dilution curves (**b**) of different greening tree species. The height of the purple bars in (**a**) represents the extent of EMF infection in the greening tree species.  represents *Cedrus deodara* in the roadside green belts;  represent *Cedrus deodara* in the park roadside green belts;  represent *Pinus massoniana* in the park roadside green belts;  represent *Salix babylonica* in the park roadside green belts.

In the two UGSHs, significant differences (*P* < 0.05) (Fig. 1a) in EMF infestation rates were observed among three greening tree species; EMF infestation rate of *S. babylonica* in park green belts was the lowest, whereas no significant difference (*P* > 0.05) in the *C. deodara* EMF infestation rate was observed. The EMF infection rates were as follows: *P. massoniana* in parks (54.48%) > *C. deodara* in parks (51.27%) > *C. deodara* on roads (41.3%) > *S. babylonica* in parks (36.97%).

### EMF community composition and structure

EMF OTUs (Table 1) was as follows: *P. massoniana* in parks (31) > *C. deodara* in parks (23) > *C. deodara* on roads (13) > *S. babylonica* in parks (9), which indicated that EMF richness was obviously different among the diverse greening trees in the different UGSHs. EMF species dilution curve (Fig. 1b) showed that with an increase in the sampling amount, the number of EMF OTUs gradually increased but did not tend to stabilize, which indicated that the sampling amount did not fully or accurately reflect EMF diversity in the study sites, suggesting large spatial variation in the EMF community in UGSHs. Therefore, increasing the number of EMF samples in subsequent related investigations is necessary.

**Table 1:** Ectomycorrhizal fungi colonizing greening tree species root from different green belts association habitats were identified based on morphotyping and sequencing of the internal transcribed spacer (ITS) rDNA.

Number	OTUs	Genbank ID	Sequence length/bp	EMF hosts
1	*Amphinema* sp.1	PQ049667	645	▽△
2	*Amphinema* sp.2	PQ049668	682	▽
3	*Archaeorhizomyces borealis*	PQ049669	530	▽
4	Basidiomycota sp.	PQ049670	680	▽
5	*Cenococcum geophilum*	PQ049671	511	▽○
6	*Cenococcum* sp.1	PQ049672	570	△
7	Ceratobasidiaceae sp.	PQ049673	697	▽
8	*Clavulicium delectabile*	PQ049674	670	▽
9	*Clavulina* sp.	PQ049675	672	△
10	*Clavulina* sp.1	PQ049676	618	▽
11	*Clavulina* sp.2	PQ049677	706	▽
12	Clavulinaceae sp.1	PQ049678	672	▽
13	*Cortinarius* sp.	PQ049679	522	▽
14	*Cortinarius* sp.1	PQ049680	634	△
15	Dothideomycetes sp.	PQ049681	542	△
16	Eurotiomycetes sp.1	PQ049682	644	▲
17	*Helvellosebacina* sp.	PQ049683	640	△
18	*Hypomyces luteovirens*	PQ049684	684	▽
19	*Ilyonectria macrodidyma*	PQ049685	584	△
20	*Ilyonectria* sp.1	PQ049686	574	△
21	*Inocybe pseudoreducta*	PQ049687	716	○
22	*Lactarius akahatsu*	PQ049688	597	○
23	*Lactarius hatsudake*	PQ049689	597	○
24	*Lactarius kesiyae*	PQ049690	762	▽
25	*Lactarius salmonicolor*	PQ049691	760	▽
26	*Lophiostoma corticola*	PQ049692	552	▲
27	*Massarina* sp.1	PQ049693	554	▲
28	*Oidiodendron citrinum*	PQ049694	540	▲△
29	*Oidiodendron maius*	PQ049695	576	▲△
30	*Oidiodendron* sp.1	PQ049696	585	▲△
31	*Oidiodendron* sp.2	PQ049697	599	△
32	*Otidea bicolor*	PQ049698	648	▽
33	*Otidea bufonia*	PQ049699	672	▽
34	*Otidea subpurpurea*	PQ049700	647	▽
35	*Pseudotomentella* sp.1	PQ049701	758	△
36	*Russula brevispora*	PQ049702	608	▲○
37	*Russula catillus*	PQ049703	648	▽△
38	*Russula cremicolor*	PQ049704	716	▽△
39	*Russula indocatillus*	PQ049705	701	▽
40	*Russula* sp.1	PQ049706	585	▽
41	*Sebacina dimitica*	PQ049707	636	▽
42	*Sebacina incrustans*	PQ049708	534	▽
43	*Sebacina* sp.	PQ049709	652	▽
44	*Sebacina* sp.1	PQ049710	537	▽○
45	*Sebacina sparassoidea*	PQ049711	652	△
46	*Thelephora* sp.1	PQ049712	653	○
47	Thelephoraceae sp.1	PQ049713	685	△
48	Thelephoraceae sp.2	PQ049714	584	▽
49	*Tomentella* sp.	PQ049715	695	▽
50	*Tomentella* sp.1	PQ049716	683	▽
51	*Tomentella* sp.2	PQ049717	683	▽
52	*Tomentella* sp.3	PQ049718	698	△
53	*Tomentella* sp.4	PQ049719	686	○
54	*Tomentella stuposa*	PQ049720	686	▽○
55	*Trichophaea* sp.	PQ049721	613	▲
56	*Trichophaea* sp.1	PQ049722	595	▲△
57	*Trichophaea* sp.2	PQ049723	614	▲△
58	*Tylospora* sp.	PQ049724	463	▽
59	*Venturia* sp.1	PQ049725	591	△
60	*Wilcoxina* sp.	PQ049726	616	▲
61	*Wilcoxina* sp.1	PQ049727	617	▲△
62	*Wilcoxina* sp.2	PQ049728	611	▲△

Note: ▲ represents the *C. deodara* on roads; △ represents the *C. deodara* in parks; ▽ represents the *P. massoniana* in parks; ○ represents the *S. babylonica* in parks.

**Fig. 2:**
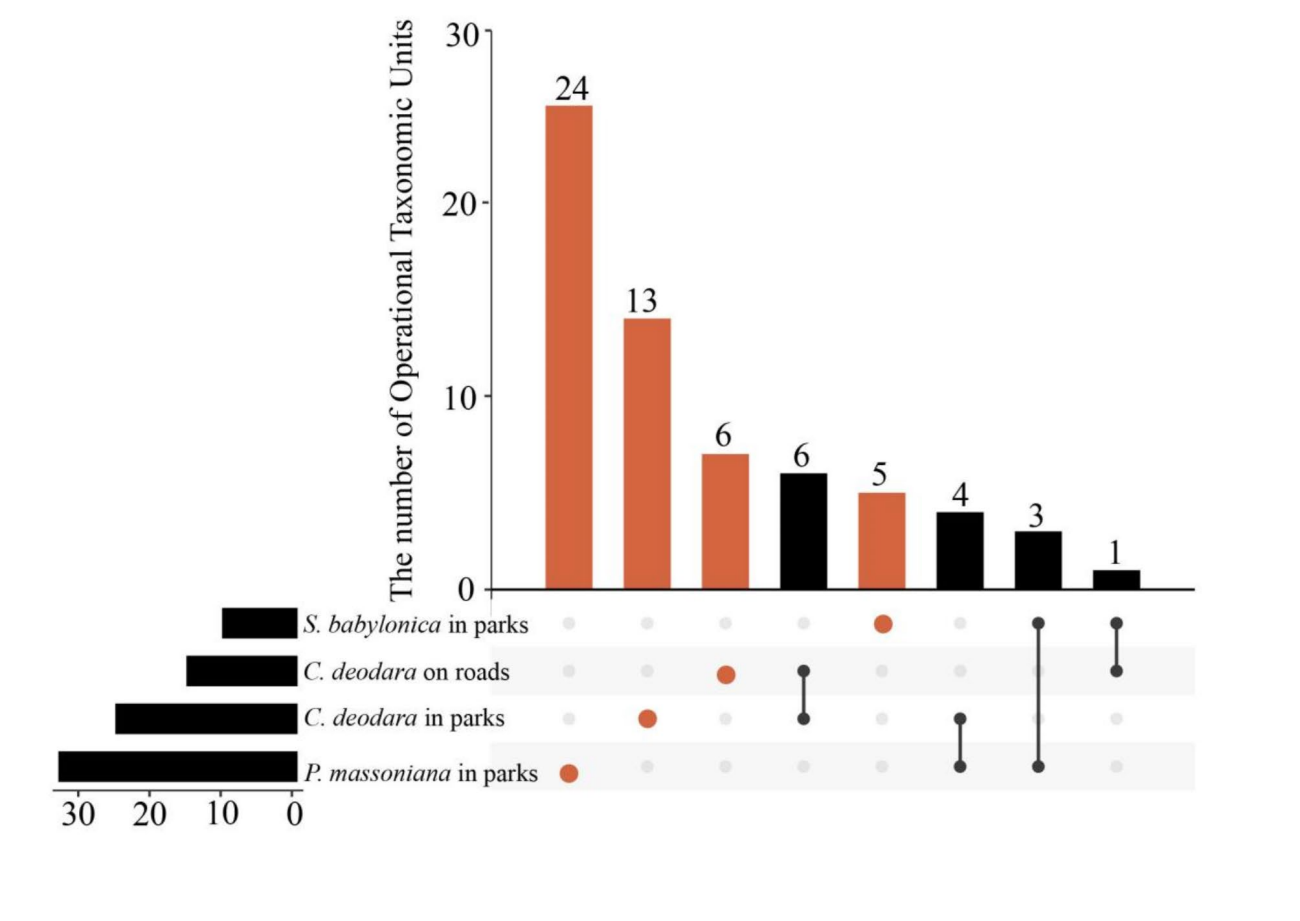
UpSet Venn diagram of Ectomycorrhizal fungi in different greening tree species. Orange-red dots and the height of the column represent the corresponding tree species and the number of unique Operational Taxonomic Units (OTUs) occupied, respectively. The line connected by the black dots indicates that the tree species corresponding at the two endpoints share common OTUs, while the height of the black column represents the number of common OTUs between the corresponding two greening tree species.

**Fig. 3:**
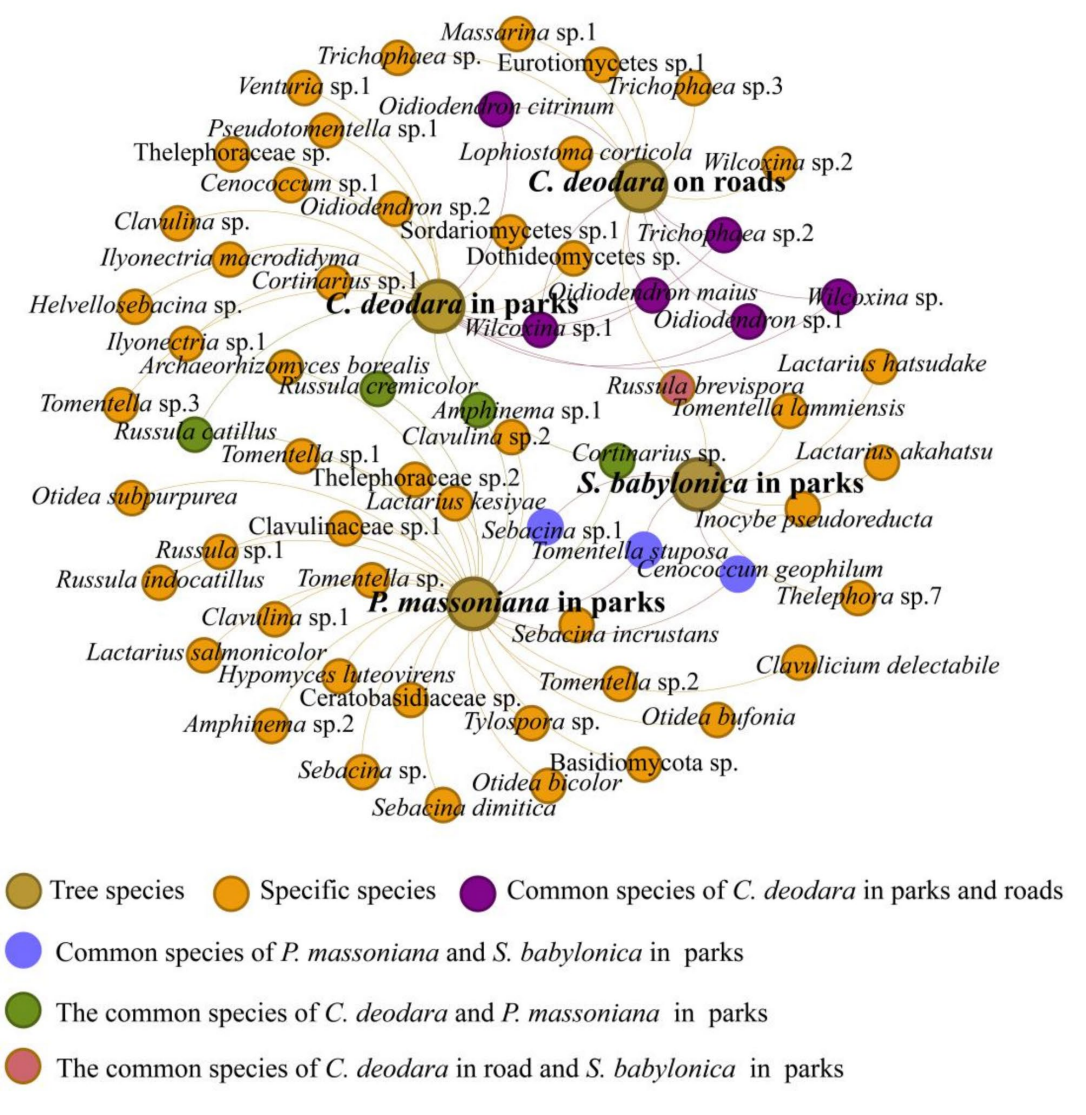
Co-occurrence network of greening tree species ectomycorrhizal fungi communities in different green belt.

EMF community in each greening tree showed that the number of specific OTUs was higher than that of common OTUs (Fig. 2), and the common OTUs were mainly *Oidiodendron*, *Wilcoxina*, and *Russula* (Fig. 3). No common OTUs were observed among the three greening trees, including *C. deodara* and *S. babylonica* in the park green belt.

**Fig. 4:**
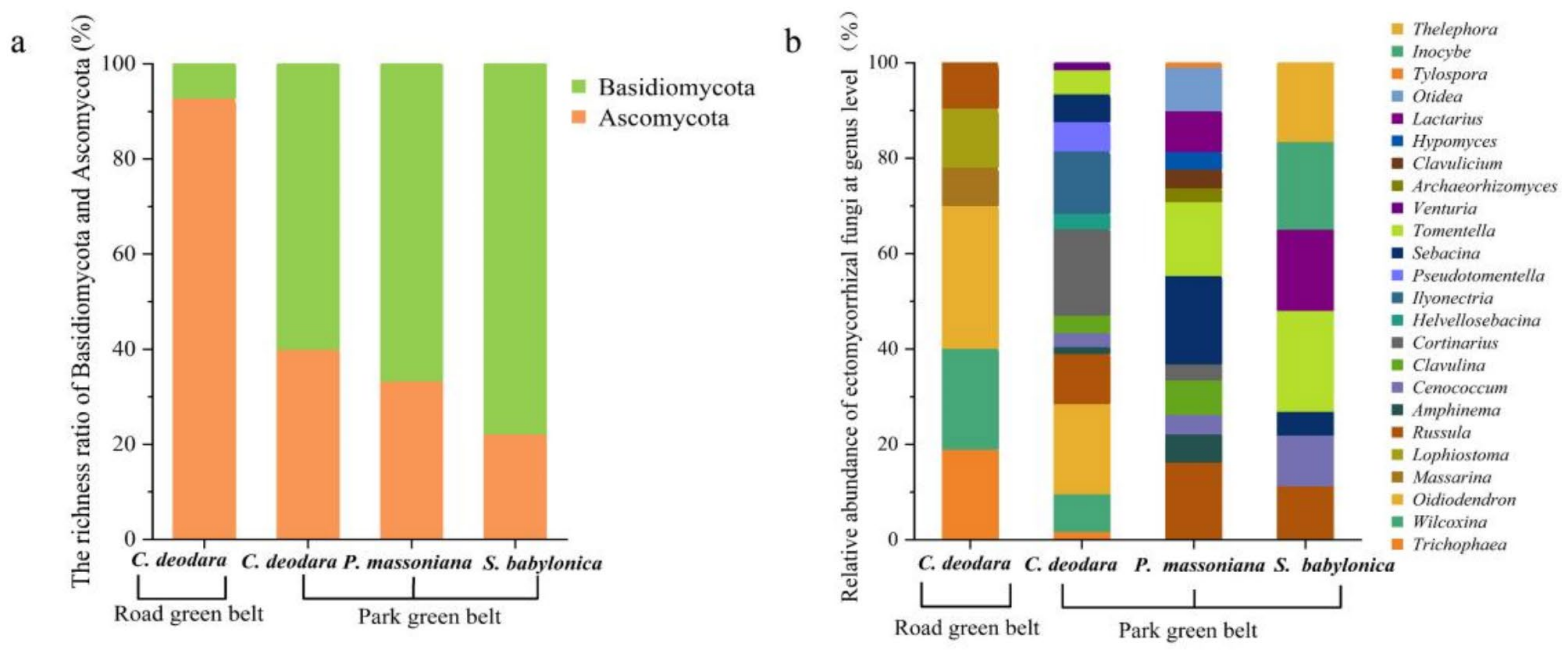
Ratios of Ascomycota to Basidiomycota fungal phylum (**a**) and relative abundance at the ectomycorrhizal fungi (EMF) genus level (**b**) in different greening tree species. The small squares of different colors represent the classification of EMF at the phylum (**a**) and genus levels (**b**) with each color corresponding to the color of the column in the figure. The height of the corresponding color column represents the richness or relative abundance of the EMF in the phylum or genus in the greening tree species.

Ascomycota and Basidiomycota were the dominant phylum in the roadside and park green belts, respectively (Fig. 4a). The EMF community structures in different UGSHs differed significantly (Fig. 4b). *Trichophaea*, *Wilcoxina*, and *Oidiodendron* were the dominant genus in roadside green belts. *Tomentella*, *Russula*, and *Genocococcum* were the dominant genus in park green belts. Both *Wilcoxina* and *Oidiodendron* were the most common dominant genus of *C. deodara* in the two UGSHs but were more abundant in the roadside green belt (Fig. 4a).

### EMF community diversity and similarity

The EMF diversity (Table 2) among greening trees was as follows: *P. massoniana* in parks > *C. deodara* in parks > *C. deodara* on roads > *S. babylonica* in parks. This shows that UGSHs disturbance tends to reduce EMF diversity in *C. deodara*, and EMF host plants might have an important influence on EMF diversity. The Jaccard and Sørenson similarity indices of the EMF communities among different host plants was low, and *C. deodara* and *S. babylonica* EMF communities differed (Table 3).

**Table 2:** EMF diversity of urban greening tree species.

Plots	EMF hosts	OTUs	Shannon index	Simpson index	Pielou index
Roadside green belt	*C. deodara*	13	2.57	0.92	0.97
Park green belt	*C. deodara*	23	3.18	0.96	0.99
	*S. babylonica*	9	2.20	0.89	0.96
	*P. massoniana*	31	3.43	0.97	0.98

**Table 3:** The Jaccard (lower left) and Sørenson (upper right) similarity index of EMF community of greening tree species in different UGSHs.

Tree species for parks and roads greening habitat	Similarity index			
	*C. deodara* on roads	*C. deodara* in parks	*P. massoniana* in parks	*S. babylonica* in parks
*C. deodara* on roads		0.33	0.00	0.09
*C. deodara* in parks	0.20		0.15	0.00
*P. massoniana* in parks	0.00	0.08		0.13
*S. babylonica* in parks	0.05	0.00	0.08	

## Discussion

EMF is highly sensitive to soil nutrient and pollutant levels^46^. EMF plays a vital role in UGSHs and serves as an indicator of environmental quality. In this study, 62 EMF OTUs related to the two UGSHs were identified in three greening tree species. The decline in EMF OTU number showed the following pattern: *P. massoniana* in parks (31) > *C. deodara* in parks (23) > *C. deodara* on roads (13) > *S. babylonica* in parks (9). Among these, EMF OTU richness of *P. massoniana* was the highest, which might be related to the fact that *P. massoniana* is an native tree in China with relatively higher EMF richness (76 ~ 138 OTUs)^38^ in natural forest habitats. EMF richness in *S. babylonica* and *C. deodara* was lower, which is consistent with previous reports (*S. babylonica* and *C. deodara* had 7 and 19 EMF OTUs, respectively^40,46^). In addition, the species dilution curve of the number of samples (Fig. 1b) showed that the sampling amount in this study was insufficient to estimate the true EMF richness of each greening tree species. Therefore, further studies with adequate greening tree species sampling volumes in different UGSHs are required.

Basidiomycota richness in park green belts was significantly higher than that in roadside green belts, which might be due to the higher forest coverage and diversity in the park^47^, resulting in plant litter rich in lignin and cellulose. Basidiomycota can be effectively degraded and utilized to meet their own growth and reproduction needs^48^. This reflects the dominant position and important role of Basidiomycota in these habitats. Ascomycota richness in the roadside green belt was higher than that of Basidiomycota, which is consistent with *P. sylvestris* EMF diversity in sandy land^49^. Ascomycota EMF species is more adaptable to various environmental stresses^50^ and is likely the reason for *Wilcoxina* and *Trichophaea* dominance. In this study, EMF community composition was different among the different greening tree species. Habitats with high plant richness drives EMF richness^25^. EMF community structures in park green belts with higher plant diversity were more complex than those in roadside green belts. Although there are more common EMF among greening tree species at the genus or higher classification level, the OTU level is host-specific^51,52^. The spatial homogeneity of soil and vegetation increases with tourism disturbance^53^. Therefore, tourist disturbances might promote *C. deodara* and *P. massoniana* EMF community composition similarity at the genus level (seven common genus). *Russula* richness negatively correlated with the soil compaction gradient^54^. However, *Russula* was widespread in this study, and its richness was highest in *P. massoniana*, indicating that *P. massoniana* in the rhizosphere soil was less compacted and disturbed by tourist activities. The relative abundance of *Wilcoxina* and *Oidiodendron* in the roadside green belt was significantly higher than that in the park *C. deodara*, probably owing to enhanced adaptability to stressful environments, becoming the dominant species in this habitat.

Studying the EMF species diversity in urban greening trees enhance the public understanding of the ecological interactions between urban and natural environments. Such research can also aid in managing and improving urban ecological quality, and promote the diversity of ecological functions^55^. EMF diversity is mainly affected by the host plant type and soil environment^8^. EMF diversity in the park green habitat was *P. massoniana* > *C. deodara* > *S. babylonica*, and EMF diversity in park green belts was higher than that in roadside green belts, which might be due to the more complex composition and structure of vegetation communities in parks^33^ with higher diversity of cultivated non-native species and frequent conservation management (including regular fertilization, tillage, and watering). These human disturbances provide heterogeneous conditions for the greening tree species. The geographical environment and soil matrix of the parks are different and are also affected by the root exudates of adjacent plants^56^. This might selectively affect specific microbial communities in the rhizosphere of plants^57^, indirectly increasing nutrient availability for EMF, providing a broader niche for EMF growth and diversity, and shaping EMF diversity. Additionally, diversity was associated with high plant species richness^58^. A small number of unique EMF groups were observed in the non-native host plants *C. deodara*. This result is likely attributed to the wide compatibility between non-native host plants and native EMF^59,60^. Notably, EMF is the only group that tends to lose diversity owing to changes in urban land use^61^. In our study, *C. deodara* exhibited varying EMF diversity across different habitats owing to differences in land use and management patterns. This result suggests that the habitats conversion of host plants may be an important driving factor for the loss of EMF diversity. However, EMF in roadside green belts might be limited by other related factors such as single vegetation species (only *C. deodara*), shallow roots, nitrogen deposition caused by automobile exhaust emissions, and changes in temperature and humidity conditions^62,63^, which lead to EMF growth inhibition. *C. deodara* in the roadside greening habitat requires more frequent fertilization and irrigation, which reduces the dependence of plants on the absorption of nutrients and water by mycorrhizal fungi^64,65^. With the increase in soil compaction and the serious impact of human activities, the soil carbon deposition rate and the soil animals’ species (for example earthworms) content are affected, resulting in decreases in EMF diversity and host root infestation rate of *C. deodara* in roadside greening habitats^38^. In this study, the similarity of EMF communities among different greening tree species was low, due to the construction of EMF community dependence on both random and deterministic processes, and the diffusion limitation in the random process is the primary influencing factor; that is, the geographical distance hinders the diffusion of fungal propagules (spores and hyphae)^35^.

## Conclusions

Overall, our study demonstrated significant differences in the composition and diversity of EMF communities among different greening tree species in two UGSHs. In addition, EMF diversity in the same greening tree species (*C. deodara*) was significantly affected in different UGSHs. UGSHs are urban functional lands and important habitats for urban biodiversity maintenance. Further studies that examine EMF diversity changes of other greening tree species from different perspectives of habitat interference are required to provide a scientific basis for healthy development of UGSHs ecology and the development of EMF agents for urban greening trees management.

## Materials and methods

### Study area

The study area is located in Huaxi District (106° 27–106° 52′ E, 26°11′–26° 34′ W), Guiyang City, Guizhou Province, China, which terrain is mainly mountainous and hilly, located in the east of Yunnan-Guizhou Plateau and the middle of Miaoling Mountains and belonged to the typical karst landform area. The area belongs to subtropical monsoon humid climate zone, the average annual rainfall approximately is 1178.3 mm, the average annual temperature is 15.7 °C, and the soil type is yellow. Zone vegetation type is evergreen broad-leaved mixed and coniferous forest. The tree species include *P. massoniana*, *C. deodara*, *S. babylonica*, *Camphor* and *Ginkgo*.

### Sample collection and processing

In April 2023, the following tree species and sample plots were selected for analysis: three greening tree species in the Huaxi park green belt–*C. deodara*, *P. massoniana*, and *S. babylonica*, and one species in the roadside green belts of Jiaxiu South Road–*C. deodara*. The site conditions information was recorded and presented in Table 4. In the survey site, a total of 40 root samples were collected, 10 healthy host plants with relatively uniform diameters at breast height and separated by a minimum spatial distance of 3 m were randomly selected as sampling objects. For each greening tree, mycorrhizal root tips were collected using a root-cutting knife within a 1–1.5 m radius around the base of the tree trunk. Moreover, samples 15–20 cm in length from a 0–20 cm deep soil layer in two directions were traced from the trunk of the selected trees. Each root sample was placed in a plastic bag, stored on ice at 4 °C and transported to the laboratory. In the laboratory, samples were combined with dry silica gel and stored in a refrigerator at 4 °C until further analysis.

The collection of plant material in our study is licensed. This materials not stored in a publicly available herbarium.

**Table 4:** Basic information of different greening tree species in the plot.

Plots	EMF hosts	Altitude	Longitude	Latitude	Diameter	Height
Roadside green belt	*C. deodara*	1093	106° 39′ 39″ E	26° 27′ 4″ N	16.48	7.57
Park green belt	*C. deodara*	1098	106° 40′ 8″ E	26° 26′ 1″ N	20.16	8.24
	*S. babylonica*	1088	106° 40′ 5″ E	26° 26′ 9″ N	19.98	6.97
	*P. massoniana*	1078	106° 40′ 29″ E	26° 26′ 37″ N	20.89	9.64

### Morphological and molecular identification of EMF

The root samples were extracted with tweezers and cut into 3–5 cm segments using scissors. After being washed repeatedly with tap water, the samples were transferred into a clean Petri dish with a small amount of water. Under the stereomicroscope (Mac Audi SMZ-171, Xiamen, China), the morphological classification was based on characteristics such as shape, size, branching, color and presence or absence of mycelium in mycorrhizal root tips^66^. The morphology and quantity of the mycorrhizal roots were recorded and photographed. Two to three root tips of each mycorrhizal were selected, placed into a centrifuge tube containing 200 µL of sterile water (3 replicates), and stored in a -20 °C freezer. Subsequently, two or three robust root tips were selected for extraction for species identification^67^. EMF DNA was extracted by modified cetyltrimethyl ammonium bromide (CTAB) method. The primers ITS1-F (5′-CTTGGTCATTTAGAGGAAGTAA-3′) and ITS4 (5′-TCCTCCGCTTATTGATATATATGC-3′) were used to amplify the polymerase chain reaction (PCR) products. The PCR system volume was 25 µL^68^. The PCR reaction conditions were as follows: initial denaturation at 94 °C for 3 min; denaturation at 94 °C for 30 s; annealing at 55 °C for 30 s; and extension at 72 °C for 60 s. This cycle was repeated for a total of 34 cycles, followed by a final extension at 72 °C for 10 min. All PCR amplifications were analyzed using 2 µL of the sample through 1% agarose gel electrophoresis. After passing the test, the amplified products were sent to Sangon Bioengineering (Shanghai, China) Co., Ltd. for sequencing.

### Data processing

The obtained DNA sequence was analyzed using Chromas (https://technelysium.com.au), and the low-quality sequence ends were trimmed and corrected. The two effective sequences of the same PCR product were spliced using SeqMan (https://www.dnastar.com). To preliminarily identify the relevant species, the spliced DNA sequence was compared against the National Center for Biotechnology Information (NCBI) (https://www.ncbi.nlm.nih.gov/genbank/) database using BLAST, with a 97% sequence similarity threshold for OTUs classification. Then, phylogenetic trees were constructed using the neighbor-joining method in MEGA-X software (https://www.megasoftware.net), with a bootstrap value of 1000. Phylogenetic analysis was carried out to further determine its OTUs. OriginPro 2022 (https://www.originlab.com) was used to draw the cumulative curve of OTU sampling number and the histogram of mycorrhizal infestation rate, and the relative abundance diagram for the phylum and genus levels was drawn. Co-occurrence network analysis of EMF was conducted at the OTU level using Gephi 0.10.2 software (https://gephi.org), and the diversity index of EMF was calculated with R version 4.3.2. (https://cran.rstudio.com). The Jaccard and Sørenson indexes were used to evaluate the EMF diversity among host plants. The simplified calculation formula is as follows:

$$J = \frac{f}{(a + b - f)};\quad S = \frac{2f}{{(a + b)}}$$

J and S represents Jaccard and Sørenson indexes, respectively; a and b represent two sets, respectively, and f represents the intersection between the two sets “a” and “b”. In this study, a and b refer to two greening tree species, respectively, and f represents the OTUs shared between the two greening tree species.

## Electronic supplementary material

Below is the link to the electronic supplementary material.

Supplementary Material 1

## Data Availability

Sequence data that support the findings of this study have been deposited in the National Center for Biotechnology Information (NCBI) with the primary accession code SUB14603063.

## Code availability

The code used in this study is available in the supplementary material published online by the paper.
