# Supplementary material for: Effects of urban green space habitats and tree species on ectomycorrhizal fungal diversity
Source: Sci Rep. 2024 Oct 25;14:25369. doi: 10.1038/s41598-024-74448-8 (PMC11511879; doi:10.1038/s41598-024-74448-8)
Supplement: Supplementary file 1 — Supplementary Material 1 [file 41598_2024_74448_MOESM1_ESM.docx]

**Supplementary materials**

**R code**

install.packages("vegan")

install.packages("picante")

install.packages("ggplot2")

library(vegan)

library(picante)

library(ggplot2)

otu=read.csv("KYHJ7.csv", header=T, row.names = 1,stringsAsFactors = FALSE, fileEncoding = 'utf-8')

otu<-t(otu)

Alpha_diversity_index <- function(x, tree = NULL, base = exp(1)) {

est <- estimateR(x)

Obs <- est[1, ]

chao1 <- est[2, ]

Shannon <- diversity(x, index = 'shannon', base = base)

Simpson <- diversity(x, index = 'simpson')

Pielou <- Shannon / log(Obs, base)

goods_coverage <- 1 - rowSums(x == 1) / rowSums(x)

result <- rbind(Obs,chao1, Shannon, Simpson,

Pielou, goods_coverage)

if (!is.null(tree)) {

Pd <- pd(x, tree, include.root = FALSE)[1]

Pd <- t(Pd)

result <- rbind(result, Pd)

}

result

}

aa <- Alpha_diversity_index(otu)

aa <- t(aa)

write.csv(aa, file = 'KYHJ7_1.csv')
